# The patient perspective: utilizing focus groups to inform care coordination for high-risk medicaid populations

**DOI:** 10.1186/s13104-017-2638-1

**Published:** 2017-07-25

**Authors:** Alex Sheff, Elyse R. Park, Mary Neagle, Nicolas M. Oreskovic

**Affiliations:** 1 Medicaid Strategies, Department of Community Health and Population Health Management, Partners HealthCare, 399 Revolution Dr., Somerville, MA 02145 USA; 2 Psychiatry-Outpatient Department, Massachusetts General Hospital/Mongan Institute for Health Policy, 50 Staniford Street, Boston, USA; 3 Harvard Medical School, Boston, MA 02114 USA; 4 Integrated Care Management Program, Massachusetts General Hospital, 125 Nashua Street, MGH, Boston, MA 02114 USA; 5 Department of Internal Medicine and Pediatrics, Massachusetts General Hospital, Boston, USA

**Keywords:** Medicaid, Care coordination, Care management, Accountable Care Organization, Focus Groups

## Abstract

**Background:**

Care coordination programs for high-risk, high-cost patients are a critical component of population health management. These programs aim to improve outcomes and reduce costs and have proliferated over the last decade. Some programs, originally designed for Medicare patients, are now transitioning to also serve Medicaid populations. However, there are still gaps in the understanding of what barriers to care Medicaid patients experience, and what supports will be most effective for providing them care coordination.

**Methods:**

We conducted two focus groups (n = 13) and thematic analyses to assess the outcomes drivers and programmatic preferences of Medicaid patients enrolled in a high-risk care coordination program at a major academic medical center in Boston, MA.

**Findings:**

Two focus groups identified areas where care coordination efforts were having a positive impact, as well as areas of unmet needs among the Medicaid population. Six themes emerged from the focus groups that clustered in three groupings: In the first group (1) enrollment in an existing medical care coordination programs, and (2) provider communication largely presented as positive accounts of assistance, and good relationships with providers, though participants also pointed to areas where these efforts fell short. In the second group (3) trauma histories, (4) mental health challenges, and (5) executive function difficulties all presented challenges faced by high-risk Medicaid patients that would likely require redress through additional programmatic supports. Finally, in the third group, (6) peer-to-peer support tendencies among patients suggested an untapped resource for care coordination programs.

**Conclusions:**

Programs aimed at high-risk Medicaid patients will want to consider programmatic adjustments to attend to patient needs in five areas: (1) provider connection/care coordination, (2) trauma, (3) mental health, (4) executive function/paperwork and coaching support, and (5) peer-to-peer support.

## Background

Care coordination programs (also referred to as care management programs) for high-risk patients are a critical component of the population based payment structures proliferating in the post-Affordable Care Act landscape. The use of multidisciplinary staff designed to support targeted patients in primary care settings by offering care coordination began as efforts in their own right, but are now central features of many Accountable Care Organizations (ACOs) and Patient Centered Medical Homes.

The Medicare Shared Savings Program created under the Affordable Care Act helped to popularize the ACO model for Medicare patients, and often included care coordination components. Most “successful” high-risk care coordination programs include Medicare patients [1]. Increasingly, Medicaid programs are now also turning to risk-based care coordination efforts such as ACOs to manage costs and improve care [2]. The result is likely to increase pressure on high-risk care coordination programs to include Medicaid patients [3].

High-risk Medicaid patients differ from high-risk medicare and private payer patients however, and care coordination programs not initially focused on the population will likely have to make significant modifications. Most importantly, Medicaid patients are much more likely to have mental health and substance use conditions. The number one hospital admission diagnosis for Medicaid patients is mood disorders [4]. Medicaid patients also face greater social challenges that can influence their access to, and experience of healthcare services including housing and food insecurity, as well as other social challenges associated with poverty [5].

### Payment and delivery reform context

A few care coordination programs have focused on Medicaid patients from their inception, with a focus on providing social supports, including housing, food, disability supports, [6] appointment navigation, coaching for chronic disease self-management strategies, patient education [7], and the supporting role of families [8]. Despite these efforts, overall cost reductions from care coordination for both Medicare and Medicaid patient populations is inconsistent, though most programs have shown reductions in ED utilization and some types of inpatient utilization [1]. In several cases, the programs actually increased utilization of outpatient care [6, 7].

### The integrated care management program

The integrated care management program (iCMP) was initiated at Massachusetts General Hospital in 2006 and broadened to the affiliated healthcare networks in 2012. This program focused on Medicare patients and embedded a nurse care coordinator within primary care practices to manage care for patients and included a supporting staff of social workers, community resource specialists, and a pharmacist. In January of 2014, the program expanded again from its initial Medicare focus to include Medicaid patients [9]. The program therefore undertook an assessment to investigate how the leading health outcomes drivers for these patients might differ. A variety of approaches were utilized including examining multiple data sources, staff interviews, and a review of other organizations’ best practices. Chief among these approaches was the use of focus groups to better understand patient perspectives on the program and their own care needs.

The purpose of this study was to use focus groups to assess the unique needs of a targeted patient population, identify gaps in support, patient-perceived barriers to care, and patient-identified opportunities for health improvement. We hypothesized that Medicaid patients enrolled in the iCMP would be able to identify barriers to care, unmet care needs, and opportunities for programmatic improvement for a medical care coordination program. The ultimate goal was to utilize this information to identify services and supports that could be helpful to the Medicaid patients in the iCMP, determine how successful the current program structure was for them, and how it might be improved.

## Methods

### Theoretical foundation

**Fig. 1:**
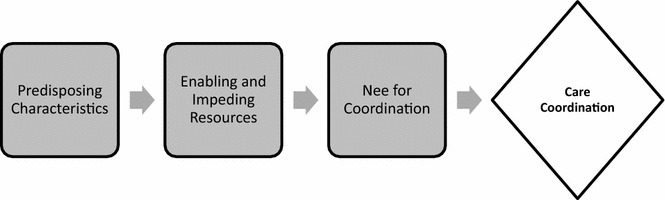
Behavior framework model

Health care utilization and successful care coordination rely both on individuals and health systems. According to the behavior framework model (BFM) (Fig. 1), a framework adapted from the Andersen behavior model by the Agency for Healthcare Research and Quality specifically for understanding the coordination of health care services, care coordination relates to three concepts: a participant’s predisposition to care coordination, the resources that enable or impede care coordination, and a participant’s need for care coordination [10]. This framework can be applied to gather insights and better understand the existing barriers, circumstances, and opportunities that will allow for the successful incorporation of Medicaid patients into a medical care coordination program. On the individual side, a patient’s predisposition to seek and accept care coordination relies on his/her knowledge, attitudes, and beliefs about care coordination, along with a patient’s psychosocial circumstances. On the resources side, there may be organizational resources that enable Medicaid enrollees to participate in care coordination (having social workers on staff, programmatic linkages to mental health or substance abuse resources), or conversely that impede participation (lack of trauma-informed care trained staff, financial barriers). Finally, with regards to the need for care coordination, this can pertain to both patient-identified (fragmented care) and systems-identified (high resource utilization) needs.

### Focus groups

We conducted two focus groups in July 2015. The focus group locations, Revere, MA and Everett, MA were selected based on a combination of a high volume of Medicaid patients within the iCMP program receiving primary care at practices in these communities, travel considerations, and space availability at the time. One hundred thirty-two patients were originally identified by programmatic review as potentially eligible based on the following criteria: English speaking, aged 18 years or older, having a Primary Care Physician associated with the medical center and receiving primary care at one of five medical practices located within the surrounding neighborhoods of where the focus groups would take place, enrolled in the iCMP, and insured by Medicaid. Of these 132 patients, 100 were found to meet all eligibility criteria. The list of eligible 100 patients was then reviewed by the patients’ primary care physicians and nurse care coordinator for appropriateness to participate in the study, based on the patients’ medical conditions and psychosocial profiles and likelihood of attending and actively participating in a focus group. Sixty-two of the 100 patients were deemed eligible for the study. Of these 62 patients, 13 declined participation, 25 were not able to be reached, and 24 expressed interest and were invited to participate, with 13 ultimately participating in the focus groups (6 and 7 participants per focus group, respectively).

Written informed consent was obtained from all individual participants prior to the focus groups. Participants received thirty-five dollars as remuneration for their participation. This study was approved by the Partners HealthCare Institutional Review Board. Focus groups lasted 60 min and were facilitated by two of the study team members (AS, MN). Written notes were recorded during and immediately following the focus groups, and focus groups were audio recorded and transcribed verbatim.

After establishing a research question, a discussion guide was developed de novo informed by the behavior framework model and the literature on Medicaid populations and with input from experts in care management and qualitative research methods. The guide was reviewed for face validity by care coordination experts (MN, NMO) and a qualitative methods expert (ERP), and pilot tested among colleagues. The guide contained questions on care needs, provider engagement, and gaps in services, open-ended questions with prompts and follow-up probes were used to collect qualitative data. Two study team members (AS, NMO) independently reviewed and manually coded the focus group transcripts and notes using inductive content analysis to identify common themes. Agreement on themes was achieved by consensus during a group session with the coders. Two separate reviewers (EP and a graduate student) subsequently reviewed a random sample of eleven focus group participant quotes and were asked to match each quote to one of the identified themes, with 100% agreement.

## Results

The focus groups identified areas where iCMP care coordination efforts were having a positive impact, as well as areas of unmet needs among the Medicaid population. Six themes emerged in three groupings: In the first group (1) traditional medical care coordination efforts, and (2) provider communication largely presented as positive accounts of assistance, and good relationships with providers, though participants also pointed to areas where these efforts fell short. In the second group (3) trauma histories, (4) mental health challenges, and (5) executive function difficulties all presented challenges faced by high-risk Medicaid patients that would likely require redress through additional programmatic supports. Finally, in the third group, (6) peer-to-peer support tendencies among patients suggested an untapped resource for care coordination programs. The six themes along with exemplary quotations are provided in Table 1. The six identified themes also aligned nicely and can be explained within the context of the behavior framework model. Three of the six identified themes represented *predisposing characteristics* (trauma histories, mental health challenges, executive function difficulties), two of the themes discussed *enabling resources* (provider communication, existing medical care coordination efforts), and one theme identified a *need for care coordination* (peer-to-peer support).

**Table 1:** Key themes and quotations

	Care coordination	Provider connection	Trauma histories	Mental health	Executive function	Peer support
Example Quote	*“My doctor was slacking on my genetics appointment and she emailed him and she was right on her game with it. “*	*“I need motivation. They [care coordinator] stay on me… Missed appointment*—*they call you and want to know why… I just love them. They’re my backbone.”*	*“My husband died, my father died, my brother died. I couldn’t go anywhere for a really long time… See I just lost my mind… I’ve’ just had loss after loss and I can’t deal.”*	*“It’s difficult to find somebody for medication management (for mood?), so I was wondering if one of the nearby [clinics] could get a medication manager, because the doctors can’t do it, or are too busy.”*	*“I cannot complete a project. I know that I have things to do, and I will look at a piece of paper, I will put my name on it, and then I have to put it away because I feel like I can’t complete it. I have attention deficit disorder, and learning disabilities including dyslexia … and I get so overwhelmed and feel totally crazy sometimes.”*	*“There are a lot of things out there that you can get help about (A domestic violence group?) It doesn’t matter if it’s here or not… There is a thing right here in the lady’s room… And there’s a number that you can call… Get yourself in a group. You’ll feel a lot better about yourself.”*

### Medical care coordination

Participants largely felt that care coordination was an area of strength. Patients reported the assistance they received from care coordinators helped them navigate the medical system and get the care they needed. They cited the challenge of figuring out which specialists they needed to see, and how to get appointments. One identified programmatic strength is having care coordinators who are familiar with and able to navigate complex health systems.

Patients also identified areas where medical care coordination efforts could be improved. Many of the Medicaid patients had few, if any face-to-face encounters with care coordinators. Patients felt that relationships were stronger with providers they saw more regularly face-to-face, such as their primary care physician. This suggested a clear gap, and lost opportunity.

### Provider connection

The importance of connection, and a peer like relationship with providers and program staff came up repeatedly. Patients praised care coordinators for providing motivation, suggesting the critical nature of their role as a coach for this patient population. This came up especially in the context of appointment reminders, as well as the sense that there was someone who was concerned for them.

There were some program design elements that did not support optimal relationship building, however. In a testament to the importance of the care coordinator–patient relationship, patients expressed frustration with automated reminders. Automated reminders from unfamiliar staff were more likely to be ignored in the context of a chaotic home life.

The importance of the provider relationship was also connected to feeling heard, and having care explained. Negative experiences around communication with providers appeared to produce lasting frustration and skepticism among patients, and had a negative impact on the doctor-patient relationship.

### Trauma

The ubiquity of trauma, and its influential and lingering effects on the Medicaid patients in the focus groups was an important and recurrent theme. Loss of a loved one was the most common example of trauma, almost unanimously related to substance use. Wide spread commonality of such experiences was notable, and likely connected to mental health challenges experienced by the patients. It was a connection that patients themselves pointed to. As one patient bluntly but clearly explained: “when I lost my son thirteen years ago, I lost my mind.”

Several patients also reported domestic violence, with lasting impact. One woman alluded to her experience explaining: “I went through a big traumatic thing for twenty years… No, I can’t talk about it because the triggers are so awful that I would end up in the hospital.” These findings suggest the importance of having trained staff capable of providing trauma-informed care.

### Mental health

Mental health challenges more broadly were also prevalent among patients participating in the focus groups. While not unexpected, their importance lies in the most commonly reported challenges. Anxiety was severe and widespread, and many patients described instances when it was triggered while obtaining care. The experience of claustrophobia, triggered by waiting alone in an exam room, was common. This led, at times, to verbal conflicts with practice staff, and potential damage to the provider-patient relationship and the patient’s investment in their own care. Patients also expressed difficulty in their efforts to obtain medication management support, specifically with regards to mental health medications.

### Executive function

Many patients expressed difficulties with task organization and completion, reporting struggles consistent with executive function deficits. While the definition of ‘executive function’ is complicated and often serves as a catch-all term for complex cognitive processes, such disorders are commonly diagnosed by neuropsychologists in relation to deficits of task-switching, as well as organizational and planning abilities. In adults this often requires anticipating future occurrences, setting goals, and selecting the appropriate steps to reach those goals [11].

In the focus groups, participants described feeling overwhelmed when faced with tasks such as navigating social and medical benefits programs, particularly those requiring paper work and filling out forms. Patients described the experience of struggling to organize themselves, and stay focused to finish multi-step tasks, ranging from going to the grocery store, to filling out benefit applications. These clear examples of executive function deficits hindered patients’ ability to get necessary care and support.

In addition to describing challenges associated with executive function, patients also shared anecdotes of assistance that helped address them. One woman who identified herself as having both ADHD and Dyslexia, shared her struggles with completing paperwork and suggested: “If I had someone to sit down and help me with it… they could ask me the question and if they wrote it down….” Another woman recounted the assistance of a Medicaid advocate, stating: “She [Medicaid advocate] would call me to remind me, about my reminder about my appointment. She would come and take me to the appointment, she took me to social security, she came to my house one day when I was flipping out and having a bad day… She took me food shopping onetime when I couldn’t get there.” Recognizing the challenges patients face regarding executive functions, and the importance that certain forms of coaching and assistance in managing a wide range of services, has critical implications for the design of care coordination programs, for this population.

### Peer-to-peer support

Another important dynamic that was observed during focus groups was peer support between participants. A sense of comradery developed early in both focus groups among patients who had never met before. Patients felt isolated in their lives. Finding others who had experienced similar challenges prompted immediate connection, and also appeared to provide positive feedback when patients perceived they were able to help one another. Participants responded positively to one another, with suggestions and encouragement upon sharing stories. Participants immediately voiced emotional support when one of them shared a difficult experience. Their own wide range of experiences with different support programs also allowed them to interject with concrete suggestions about resources others could turn to for help with a given task. Participants suggested exercise opportunities, case management services, as well as ways others could advocate for themselves, or address challenges with their provider’s offices.

## Discussion

The findings from the focus group provide important insights about the unmet needs and most effective program features for the Medicaid patient population. While the findings have direct implications for the MGH iCMP as it considers ways to improve for Medicaid patients, they also have broader lessons for any high-risk, care coordination programs seeking to support Medicaid patient populations. The program design implications associated with each of the themes from the focus groups are indicated in Table 2.

**Table 2:** Program design implications

Provider connection/care coordination	Trauma support	Mental health	Coaching and paperwork assistance	Peer-to-peer support
Enable additional avenues for care coordinator-patient connection (i.e. home visits, regular phone contact) Appointment reminders should be the responsibility of the primary member of the care coordination team Utilize non-nurse providers such as Community Health Workers to enable more continuous out of office contact	Offer direct treatment for trauma, or connect patients with external resources such as trauma and grief counseling Provide trauma informed care training to all program staff, especially those operating as primary care coordinators	Appoint additional social workers as the primary care coordinator and first point of contact for appropriate patients Incorporate psychiatric nurses into the care coordination team Require mental health and addiction training for all staff to reduce patient stigma	Consider ways to identify patients with executive function deficits Expand the community health worker role for patients identified as needing additional supports with paperwork and task completion Emphasize the use of motivational interviewing techniques for patient coaching	Incorporate peer support arrangements, including through electronic communication and social networks

Provider connection was powerful in helping patients engage in their own care, but was hampered by a lack of face-to-face interactions with care coordinators. Establishing additional avenues for care coordinators to reach patients in person-either in the office or outside of it—would help fully realize the value of the care coordinator–patient relationship. Achieving greater personalization, however, raises the question: Is dedicating time for personal outreach the best use of time for a nurse care coordinator whose strength is his/her medical expertise? Given the differing needs of the Medicaid patient population, utilizing other types of providers for patients with more socially driven needs, might be a better allocation of resources. Community health workers, in particular, are valued because of their ability to form stronger peer-to-peer relationships than the traditional provider-patient relationships in the healthcare system [12]. They may therefore be better equipped for extensive home visiting, regular phone contact, and offering a community presence.

Consistent with previous research, patient trauma triggered anxiety that remained an impediment to health improvement and appeared to drive care utilization [13]. Care coordination programs for Medicaid patients may therefore want to consider offering or connecting patients with trauma and grief counseling. The focus groups also suggested providers, including care coordinators can, and may unintentionally trigger trauma experiences jeopardizing near term goals (i.e. appointment scheduling or filling out benefit forms) and potentially worsening the patients’ mental and emotional status. Extensive literature has developed around the best practices for trauma-informed care to avoid triggering patients, and furthering trauma [14]. Trauma-informed care training for staff would therefore likely be an important investment for care coordination programs.

The benefits of behavioral health integration are well established, and with the mental health needs of the Medicaid patient population, high-risk care coordination programs role in this broader effort could include appointing more social workers as the primary care coordinator and first point of contact for appropriate patients [15]. This could help patients manage anxieties, ease friction with other providers, and increase program enrollment. Incorporating a psychiatric nurse into the care coordination team could also improve medication adherence and mental health capacity [16]. Finally, training regarding mental health and addiction can help avoid patient stigma [17].

Given the difficulties so many patients experienced with task completion, public benefits navigation, and basic paperwork, providing this population with in-person coaching assistance, could produce lasting benefits that increase long term compliance, and more sustainable positive health outcomes [18]. Identifying patients with executive function deficits is one way to triage patients and more appropriately target supports. Here too, developing or expanding roles for community health workers could be critical. As front-line team members, community health workers have been found to be effective at providing social supports for patients [12]. A heavy emphasis on the use of motivational interviewing techniques by all care coordination team members to assist patients through challenging tasks that require high levels of executive function could also be a helpful tool [19].

The instantaneous peer-to-peer connection between focus group participants and the concrete support and guidance they offered one another, suggests Medicaid patients may be an untapped resource for each other. While evidence of the cost effectiveness of paid peer-support programs is not conclusive, some forms of peer support for mental health patients have been shown to reduce readmission rates [20]. Therefore, care coordination programs should consider incorporating peer support arrangements, including through electronic communication and social networks.

The study has several limitations. Focus group participants were mostly white, female, and older than the overall Medicaid population, and our findings may therefore not be generalizable to all Medicaid populations. Focus groups were conducted in English which negatively impacted the participation of non-English speaking patients. Younger eligible patients were more likely to be working, and therefore less likely to be available to participate even though the focus groups were held in the evenings to accommodate potential work schedules. Despite these limitations, given the paucity of information available on high-risk Medicaid patients and their increasing enrollment into risk contracts and care coordination programs, the findings from this study provide key insight into understanding the unique needs, attitudes, beliefs, and drivers of healthcare utilization risk in this population.

## Conclusion

This research utilized focus group studies to report on the critical perspectives of high-risk, Medicaid patients—the challenges they face, and their experience in the iCMP. The findings have implications for the many high-risk care coordination programs now focusing on Medicaid patients. The focus groups pointed to the strengths of programs like iCMP regarding medical care coordination and provider connection. However, they also highlight the need to increase patient contact for this population through a wider range of staff. The findings demonstrate a clear need to focus on building capacity to support patients with trauma histories and mental health needs, and suggest the benefits of leveraging peer support opportunities. To accomplish these goals, programs may want to consider additional home visits and a greater community presence, the inclusion of community health workers, and social workers, as well as training to help staff provide trauma informed care, and avoid stigma. As more states look to population based payment structures like ACOs to manage Medicaid costs, the stakes for high-risk care coordination programs could not be higher, and the perspectives of patients could not be more important.

## Abbreviations

ACO: Accountable Care Organization
iCMP: integrated care management program
BFM: behavior framework model
MGH: Massachusetts General Hospital
ADHD: attention deficit hyperactivity disorder

## References

[1] Hong CS, Siegel AL, Ferris TG (2014). Caring for high-need, high-cost patients: what makes for a successful care management program?. Issue Brief (Commonw Fund).

[2] Kocot SL, Dang-Vu C, White R, McClellan M (2013). Early experiences with accountable care in Medicaid: special challenges, big opportunities. Popul Health Manag.

[3] Government Accountability Office (2015). MEDICAID: a small share of enrollees consistently accounted for a large share of expenditures.

[4] Lopez-Gonzalez L, Pickens GT, Washington R, Weiss AJ (2014). Characteristics of Medicaid and Uninsured Hospitalizations, 2012.

[5] Crawford M, McGinnis T, Auerbach J, Golden K (2015). Population health in Medicaid delivery system reforms.

[6] Sandberg SF, Erikson C, Owen R, Vickery KD, Shimotsu ST, Linzer M, Garrett NA, Johnsrud KA, Soderlund DM, DeCubellis J (2014). Hennepin Health: a safety-net accountable care organization for the expanded Medicaid population. Health Aff (Millwood).

[7] Bell JF, Krupski A, Joesch JM, West II, Atkins DC, Court B, Mancuso D, Roy-Byrne P (2015). A randomized controlled trial of intensive care management for disabled Medicaid beneficiaries with high health care costs. Health Serv Res.

[8] Jackson CT, Trygstad TK, DeWalt DA, DuBard CA (2013). Transitional care cut hospital readmissions for North Carolina Medicaid patients with complex chronic conditions. Health Aff (Millwood).

[9] Kodner DL (2015). Managing high-risk patients: the Mass General care management programme. Int J Integr Care.

[10] McDonald KM, Sundaram V, Bravata DM, Lewis R, Lin N, Kraft SA, McKinnon M, Paguntalan H, Owens DK. Closing the quality gap: a critical analysis of quality improvement strategies (Vol. 7: Care Coordination). 2007, Technical Reviews, No. 9.7. 20734531

[11] Roth RM, Isquith PK, Gioia GA. Behavior rating inventory of executive function (BRIEF)-A (adult). PAR; 2013.

[12] Rosenthal EL, Brownstein JN, Rush CH, Hirsch GR, Willaert AM, Scott JR, Holderby LR, Fox DJ (2010). Community health workers: part of the solution. Health Aff (Millwood).

[13] Harris M (1994). Modifications in service delivery and clinical treatment for women diagnosed with severe mental illness who are also the survivors of sexual abuse trauma. J Ment Health Adm.

[14] Davis R, Maul A (2015). Trauma-informed care: opportunities for high- need, high-cost medicaid populations.

[15] Archer J, Bower P, Gilbody S, Lovell K, Richards D, Gask L, Dickens C, Coventry P (2012). Collaborative care for depression and anxiety problems. Cochrane Database Syst Rev.

[16] Nursing Times. http://www.nursingtimes.net/medication-management-for-people-with-a-diagnosis-of-schizophrenia/205851.fullarticle.

[17] Knaak S, Modgill G, Patten SB (2014). Key ingredients of anti-stigma programs for health care providers: a data synthesis of evaluative studies. Can J Psychiatry.

[18] Davis K. To lower the cost of health care, invest in social services. Health Aff (Millwood). 2015.

[19] Tahan HA, Sminkey PV (2012). Motivational interviewing: building rapport with clients to encourage desirable behavioral and lifestyle changes. Prof Case Manag.

[20] Sledge WH, Lawless M, Sells D, Wieland M, O’Connell MJ, Davidson L (2011). Effectiveness of peer support in reducing readmissions of persons with multiple psychiatric hospitalizations. Psychiatr Serv.

